# Clinical, epidemiological, genetic, and electrophysiological characteristics of transthyretin familial amyloid polyneuropathies in Israel

**DOI:** 10.1186/1750-1172-10-S1-O5

**Published:** 2015-11-02

**Authors:** Menachem Sadeh, Avi Livneh, Michael Arad, Alexander Lossos

**Affiliations:** 1 Wolfson Medical Center, Department of Neurology, 58100, Holon, Israel; 2 Sheba Medical Center, Department of Medicine F, 52621, Ramat Gan, Israel; 3 Sheba Medical Center, Institute of Cardiology, 52621, Ramat Gan, Israel; 4 Hadassah Medical Center, Department of Neurology, 91120, Jerusalem, Israel

## Background

Only a few patients and families with transthyretin associated familial amyloid polyneuropathy (TTR-FAP) have been described by different authors in Israel. The objective of this study was to elucidate the natural history, clinical manifestations, electrophysiological features, ethnic origin and genetic findings of all the known patients with TTR-FAP in Israel.

## Methods

We reviewed the medical records of all the patients that have been reported and those who have not yet been described. We retrospectively assessed the major clinical, laboratory and genetic findings of the patients.

## Results

Seventeen patients were studied. All were Jews. Eleven were of Yemenite descent, harboring the ser77tyr mutation. Of these, seven belonged to a large 3-generation family, and each of the other four to different unrelated families. Three patients were Ashkenazi; one carried the val30met mutation, another the phe33leu mutation, and the third had two mutations on one allele: phe33Ile and gly6ser. Two patients were of Iranian origin showing val32ala mutation, and one of Tunisian origin showing the val30met mutation. Onset in most patients was in the sixth decade, presenting with sensory loss of the lower and upper limbs. About half of the patients experienced at onset pain, autonomic nervous system manifestations and demonstrated evidence of amyloid cardiomyopathy. One patient of Yemenite descent presented with amyloid cardiomyopathy without neuropathic features. Nerve conduction studies showed sensorimotor axonal neuropathy in all. Sural nerve biopsies were obtained in eight patients; two biopsies did not reveal amyloid deposit. The average course was rapid, and most patients died within 4-7 years. The cause of death was intestinal malabsorption and cardiomyopathy. Two patients of Yemenite origin underwent liver transplantation, which slowed down the disease progression.

## Conclusion

TTR-FAP exists in the Israeli population and is disproportionately common among Yemenite Jews. The worldwide common mutation val30met is rare. The presence of other mutations may explain the relatively rapid course of the disease in the Israeli patients.

